# Impaired instance acquisition as a cause of the comorbidity of learning disorders in young adults

**DOI:** 10.3389/fnbeh.2025.1560362

**Published:** 2025-06-10

**Authors:** Chiara Valeria Marinelli, Giuliana Nardacchione, Marialuisa Martelli, Vincenza Tommasi, Marco Turi, Paola Angelelli, Pierpaolo Limone, Pierluigi Zoccolotti

**Affiliations:** ^1^Cognitive and Affective Neuroscience Laboratory, Department of Humanities, Letters, Cultural Heritage and Educational Studies, Foggia University, Foggia, Italy; ^2^Department of Psychology, Sapienza University of Rome, Rome, Italy; ^3^Applied Psychology Lab, University of Salento, Lecce, Italy; ^4^Department of Psychology and Education, Pegaso University, Naples, Italy; ^5^Tuscany Rehabilitation Clinic, Montevarchi, Italy

**Keywords:** dyslexia, instance learning, automatization, learning disorders, comorbidity

## Abstract

**Introduction:**

The “*instance theory of automatization*” suggests that automaticity relies on acquiring specific instances that enhance performance, preventing the slower application of procedures. It has been proposed that a low ability in instance acquisition may be the key cause of the comorbidity among learning disorders. We investigated performance on a learning task to test the hypothesis that difficulties in acquiring and consolidating instances would be linked with comorbid learning disorders.

**Methods:**

We examined the individual rate of learning of 143 young adults with typical development (32M, 111F, mean age: 20.3) and 59 with specific learning disorders (SLD; 12M and 47F, mean age: 20.9).

**Results:**

Both groups significantly reduced their response times across learning trials (following a power trend) without generalization to untrained items, indicating that learning occurred through instance acquisition. Initially, participants with SLD performed worse than the controls. However, they reduced their times by about 96 sec with practice, even though their “endpoint” (asymptote) remained slower than controls. Group differences were related to these two scaling values, not the power curve coefficient. Subsequently, we reclassified the sample into three groups based on the type of deficit: one without procedural/instance deficits (“Control” group), one with selective deficits in “procedural” tasks (“Poor procedural” group), and one with deficits in instance-based tasks (“Poor instance” group). The poor instance group not only showed deficits across all tasks requiring instance retrieval (i.e., arithmetical facts and lexical representation retrieval) but was also slower (86 s) in the learning task compared to the other groups (58 and 70 s, respectively; at least *p* < 0.01). The “Poor procedural” group behaved similarly to the “Control” group.

**Conclusion:**

Results support with the notion that a low ability to acquire and consolidate instances may contribute to the comorbidity of learning disorders.

## 1 Introduction

Developmental disorders in reading, writing, and mathematics tend to occur in association, a phenomenon known as comorbidity (Pennington, 2006; Landerl and Moll, 2010). About 40% of children with dyslexia, characterized by significant and persistent difficulties in reading accuracy and/or speed, also have low spelling skills, while 11-70% of children with dyscalculia show comorbidity with dyslexia (Moll et al., 2014). Similar results were obtained in Italian, the language assessed in the present study (Savelli et al., 2009; Trenta et al., 2009). Furthermore, it is well-known that specific learning disorders (SLD) are also associated with other developmental disorders, such as ADHD (e.g., Willcutt et al., 2019; for data on Italian: Gagliano et al., 2007; Trenta and Zoccolotti, 2012).

It has been proposed that the etiology of developmental disorders is multifactorial and that these multiple factors partly overlap (Pennington, 2006). This approach is considerably different from traditional cognitive modeling in which different learning deficits have typically been examined separately. The “*multiple deficit model*” (Pennington, 2006) proposes that there are shared processes among the disorders from the etiological, neural, and cognitive levels. Accordingly, the etiology of SLDs is multifactorial and involves the interaction between numerous risk and protective factors (genetic and/or environmental). These factors cause impairment in the normal development of neuropsychological functions, resulting in the various behavioral manifestations typical of SLDs.

The proposal by Pennington (2006) gave rise to a considerable amount of research focused on isolating the factors which may contribute to the overlap among learning and other developmental disorders (e.g., Cheng et al., 2018; Moll et al., 2019; Raddatz et al., 2017). Our research tackled this problem by examining which cognitive factors predict reading, spelling, and math performance (Zoccolotti et al., 2020a,2021b). We found some factors to selectively predict only one behavior (reading, spelling, or mathematics). By contrast, some predictors (including tests of arithmetic facts and orthographic judgment) predicted performance across all these behaviors. Considering that both tests rely on retrieving memory traces (i.e., instances) from memory, we proposed that the ability to acquire/recover instances explain the comorbidity among learning disorders.

**FIGURE 1:**
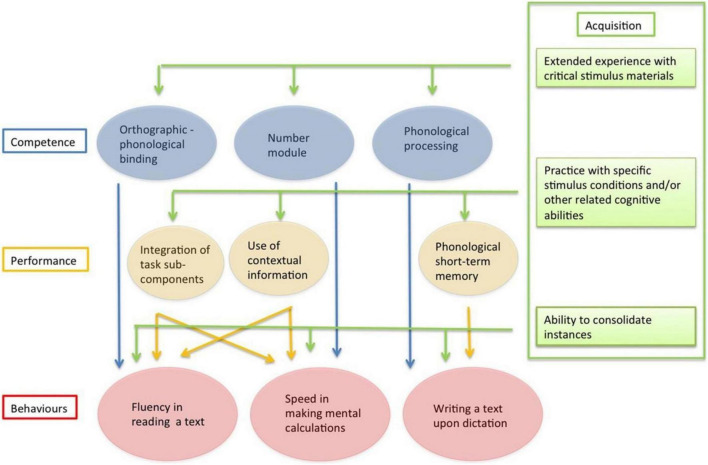
Multilevel model of learning by Zoccolotti et al. (2020b).

Based on these findings, we have proposed the “*Multilevel model of learning*” (Zoccolotti et al., 2020b). According to the model (Figure 1), different skills account for the partial independence of reading, spelling, and mathematical behaviors. Instead, comorbidity among learning disorders would be largely due to a common difficulty in acquiring and consolidating single events or “instances.”

It should be noted that the poor ability to acquire “instances” does not make reading, spelling, and making computations impossible but rather impairs the ability to do so smoothly and efficiently through automatic processing. Indeed, learning disabilities do not refer to a child’s inability to learn to read or do calculations as much as to the failure to do so fluidly and efficiently (Zoccolotti et al., 2020b). In other words, children with dyslexia can read, but their reading is stunted, labored, and not automatic due to controlled and voluntary sublexical processing (Zoccolotti et al., 2021a). Similarly, a deficit in retrieving instances in computation does not prevent the ability to perform calculations by applying computational procedures. However, it is much slower, time-consuming, and more error-prone than automatically retrieving the result from memory by direct access to arithmetic facts.

A formalization of how reference to instance learning may contribute to the development of automatization has been put forward by Logan (1988, 1992). According to his “*Instance theory of automatization*” (Logan, 1988, 1992), one initially performs a new task by learning and applying rules or an algorithm. Then, as one proceeds with extended practice (associated with formal and explicit instructions received during the school experience), through systematic repetition of the same task, the student moves from serial, slow and controlled processing by applying rules (or algorithms) to automatic processing by acquiring specific instances. Instance learning is well described by a power function (as originally proposed by Newell and Rosenbloom, 1981). This function describes a process in which performance improvements are progressively smaller with time and practice. This means that after a relatively rapid performance improvement, further improvements become progressively smaller, making the overall acquisition process relatively long (and without a defined endpoint). Note that automatization would occur at the point of the nearly flat function obtained after prolonged practice (not in the initial fast acquisition). Instance acquisition speeds up behavior and makes performance automatic (Schneider and Chein, 2003), as it leads to the gradual creation of memory traces that allow for the rapid retrieval of the solution (Logan, 1988, 1992; Compton and Logan, 1991).

This mechanism may underlie the consolidation of knowledge in reading, spelling, as well as mathematics and, if compromised, would contribute to the partial overlap between the different forms of learning difficulties. Learning to read (spell or make calculations) involves the acquisition of specific procedures. However, with extended practice, the child learns individual targets (e.g., regular, frequent words, but also irregular words such as “heart,” or the output of simple mathematical operations, such as 3 × 8 = 24), which help optimize performance.

It should be noted that there are alternative views of how the automatization of behavior is achieved. Ullman (2004) has proposed and developed (Ullman et al., 2020) the “*Procedural deficit hypothesis.*” Accordingly, failure to automatize performance occurs because of an inability or reduced capacity to acquire new procedures. This proposal heavily draws on the neurophysiological literature pointing to distinct neural circuits for procedural and declarative processes (Ullman, 2004). Children with dyslexia would be impaired in acquiring new procedures, and this basic impairment would inhibit their reading acquisition. Ullman et al. (2020) propose that a procedural deficit may underlie various developmental disorders (including developmental language disorder, motor-speech disorders, and dyslexia), thus contributing to understanding their comorbidity.

Overall, the “*Multilevel model of learning*” (Zoccolotti et al., 2020b) and the “*Procedural deficit hypothesis*” (Ullman, 2004; Ullman et al., 2020) make different predictions on the factors producing learning disorders and their frequency of association. Below, we briefly summarize the studies aimed at testing the different predictions.

The *Procedural deficit hypothesis* has given rise to a wealth of empirical research. Several studies have reported children with dyslexia to be impaired in tasks such as the serial reaction time task (e.g., Vicari et al., 2003, 2005; Howard et al., 2006; Menghini et al., 2006, 2008) or artificial grammar learning (e.g., Kahta and Schiff, 2016; Pothos and Kirk, 2004). However, several failures to replicate these findings have also been reported (e.g., Kelly et al., 2002; Rüsseler et al., 2006). Accordingly, a series of recent reviews and meta-analyses have examined this literature but reached quite discrepant conclusions (Lee et al., 2022; Lum et al., 2013; Schmalz et al., 2017; van Witteloostuijn et al., 2017; West et al., 2021). Note also that Ullman (2004) and Ullman et al. (2020) proposed a deficit in procedural learning as the likely source of comorbidity among learning and other developmental disorders; however, most studies only examined children with dyslexia, thus providing a limited test of this prediction.

The “*Multilevel model of learning*” (Zoccolotti et al., 2020b) has also been submitted to an empirical test. Based on a network study, Zoccolotti et al. (2021b) demonstrated that reading, spelling, and calculation are supported by distinct yet partially overlapping networks while still being interrelated. The abilities to retrieve arithmetic facts, spell ambiguous words, and judge the orthographic correctness of irregular fakes were the points of contact connecting reading, spelling, and calculation performances. These skills are attributable to the ability to retrieve mnestic units (i.e., instances) from memory, in keeping with the idea that the association between these three skills may be explained by the ability to acquire instances. Evidence consistent with the “*Multilevel model of learning*” (Zoccolotti et al., 2020b) also comes from studies of children with dyslexia and orthographic dysgraphia, which found that they have an orthographic lexicon consistently reduced in reading and writing (Marinelli et al., 2017, 2021). Through a “multiple single case” study examining the words known by each child, we observed specularity of performance: if the orthographic representation of a word was present in the lexicon, it was identically used in reading and spelling while, if not possessed, it was not used in either task (Angelelli et al., 2010). This finding is in keeping with the idea that comorbidity between reading and spelling is associated with reduced knowledge of lexical information, i.e., individual instances.

In a sample of typically developing children, Marinelli et al. (2021) studied the ability to acquire instances through an alphanumeric learning test and evaluated whether this ability underlies performance on the reading, writing, and computation tests based on instance retrieval. Parameters indicating the ability to acquire instances were extrapolated for each participant. A reduced ability to form and consolidate instances in the experimental test accounted for the difficulties in orthographic representations (in tests of dictation and spelling judgment with ambiguous words) and arithmetic facts (recalling from memory tables and simple calculations). The ability to acquire instances was not associated with any sublexical skills in reading or writing, with any tests based on numerical skills or with logical reasoning and working memory skills.

Overall, both the “*Procedural deficit hypothesis*” (Ullman, 2004; Ullman et al., 2020) and the “*Multilevel model of learning*” (Zoccolotti et al., 2020b) have been subjected to experimental tests. The former has generated a considerable amount of research, although with mixed results and largely confined to dyslexia; the latter has generated fewer studies but with somewhat more consistent results. Interestingly, the two hypotheses make opposite predictions as to which factors contribute to the co-occurrence among learning disorders. For the former, co-occurrence would be due to an inefficiency in procedural acquisition, for the latter, it would be due to a low ability to acquire and consolidate individual instances. We have observed that the availability of opposite predictions might help make a more stringent test on the sources of comorbidities (Marinelli et al., 2024). Such a test has not yet been carried out and is the main goal of the present research.

The present study draws on research by Marinelli et al. (2021), aiming to test the ability to acquire instances by examining a sample of college students with various SLDs. The performance of young adults with SLDs was compared to that of typically developing adults, with the hypothesis that the former would perform worse in acquiring instances. First, we compared individuals with SLDs (independent of the type of deficit) with control observers. Subsequently, we contrasted more directly the predictions derived from the “*Multilevel model of learning*” (Zoccolotti et al., 2020b) and the “*Procedural deficit hypothesis*” (Ullman, 2004; Ullman et al., 2020). The performance of young adults with selective drops in either instance-based skills (across reading, spelling, and math) or procedural skills (across reading, spelling, and math) was compared with that of young adults with no selective difficulties in either instance or procedural processing.

Note that this is a somewhat different approach to classical comorbidity studies that compare samples with one or more diagnostic categories, such as children with dyslexia vs. children with dyslexia and dyscalculia (e.g., Cheng et al., 2018; Raddatz et al., 2017). It has been observed that clinical diagnoses refer to “*complex behavioral disorders*” (Pennington, 2006) and are based on standardized tests that call on both rule-based and item-based processing (typically difficult to distinguish in this type of test) (Marinelli et al., 2024). Here, we define grouping based on consistent patterns of performance (i.e., either procedural or instance-based processing deficit) independent of the behavior domain to assess whether these components may capture comorbid differences in the learning process of a novel task.

## 2 Materials and methods

### 2.1 Participants

A total of 251 Italian college students with performance within limits on the Raven’s SPM test (Raven et al., 1998) participated in the study. Of these, data of 33 participants whose power curve fit on the Logan test had an *R*^2^ < 0.30 (*N* = 28 participants; see Data analysis) or an extreme beta value as > of 4 (*N* = 5 participants) were eliminated, resulting in a final sample of 218 participants. We relied on strict criteria to select the experimental sample. Thus, we did not consider the 16 cases with subclinical, borderline performance (i.e., failing only in one of the tests for the cognitive components assessed, e.g., one of the reading tests), resulting in a final sample of 202 participants. We did not include foreign students.

The criterion for inclusion of the group of participants with specific learning disorders was the presence of at least one reading, writing, or computation disorder on a standardized clinical test (LCS-SUA; Montesano et al., 2020). The diagnosis of dyslexia was made to participants who had pathological performance (at least *z* < −1.65) in accuracy and/or speed in reading one passage and in accuracy and/or speed in reading at least one other test (word and nonword reading). The diagnosis of orthographic dysgraphia was made to participants who had pathological performance (at least *z* < −1.65) in a passage dictation and at least one subtest of the Single Word and Nonword Dictation test. The diagnosis of dyscalculia was made to participants who had pathological performance (at least *z* < −1.65) in more than 50 per cent of the math tests and a deficit in more than one domain (calculus, arithmetic facts, and number system). See section 2.2 and Supplementary Appendix A for a description of the tests and instruments used.

The demographic characteristics and performance in reading, writing and math tasks of the resulting sample of adults with learning disorders and controls are presented in paragraph 3.1.

The study was conducted according to the principles of the Helsinki Declaration and approved by the Ethics Committee of Psychological Research of the Humanities Department of the University of Foggia (Prot. 011/CEpsi of 2/5/23). Participants were tested for about 2 h (two sessions of 1 h each) in the Cognitive and Affective Laboratory of the University of Foggia.

#### 2.1.2 Re-classification of participants as a function of the procedural vs. instance-based type of deficit

The whole sample of 218 participants (including the 16 sub-clinical participants, all with *R*^2^ > 0.30) was re-classified according to the type of deficit shown on the screening tests. Specifically, participants with (1) no deficit in both procedures and instances (referred to as “Control” group); (2) a selective deficit in procedures (referred to as “Poor procedural” group); (3) a deficit in instances (referred to as “Poor instance” group) were identified.^1^

To estimate difficulties in instance-based processing, we averaged performance in tests assessing the use of instances in computation (Arithmetic facts, Montesano et al., 2020; Simple Arithmetic Facts, Tables; Marinelli et al., in preparation), reading (all conditions with irregular words of the Orthographic Judgment test, Nardacchione et al., submitted) and spelling (Irregular word subsets of the Word and nonword dictation test, Marinelli et al., in preparation; Lexical spelling, Nardacchione et al., submitted). To estimate difficulties in procedural processing, we averaged scores at numerical tests (Numbers Transcription, Reading Numbers, Number Dictation; LCS-SUA, Montesano et al., 2020; Transcription of Numbers, Find the Major (Letters), Find the Major (Digits), Marinelli et al. (2020)), sublexical reading (Non-word subset of the Word and nonword reading tests of Montesano et al., 2020 and Marinelli et al., in preparation tests; Same/different Judgment test, Marinelli et al., in preparation) and sublexical spelling (Nonwords subsets of the Word and Nonwords Dictation Test; Marinelli et al., in preparation). Tasks that might be solved with both procedures were not considered for the identification of subgroups.^2^

For inclusion in the “Poor procedural” group, participants had to have a deficit in at least one procedural skill and performance in the normal range (within one standard deviation) on the average of the instance-based tests. For inclusion in the “Poor instance” deficit group, participants had to have a performance of less than 1.5 standard deviations in the mean of all instance-based tests. Note that the participants in the “Poor instance” group may also suffer from a procedural deficit because this group included all participants with a deficit in instances present either in isolation or in association with a procedural deficit. For inclusion in the control group, participants had to have a performance within one standard deviation in both the mean of instance-based tests and in all procedural skill-based tests. Cases that did not fit into any of the above categories and had borderline performance between the two categories were not analyzed in this part of the study (*N* = 40).

The characteristics of the resulting “Poor instance,” “Poor Procedural” and Control groups are described in paragraph 3.3.1.

### 2.2 Instruments and procedures

#### 2.2.1 Reading assessment

Reading ability was examined with: Reading Comprehension (LCS-SUA Battery, Montesano et al., 2020), Text Reading (LCS-SUA Battery, Montesano et al., 2020), Word and Non-word Reading Test (LCS-SUA Battery, Montesano et al., 2020), Word and Non-word Reading with time pressure (Marinelli et al., in preparation), Lexical Decision in Articulatory Suppression Condition (LCS-SUA Battery, Montesano et al., 2020), Same/different Judgment (Marinelli et al., in preparation), and Orthographic Judgment (Nardacchione et al., submitted). Test procedures are described in detail in Supplementary Appendix A.

#### 2.2.2 Spelling assessment

Writing ability was examined with Text Dictation Tests (Montesano et al., 2020), Word Dictation Tests in Normal and Articulatory Suppression Conditions of the LCS-SUA (Montesano et al., 2020), the Word and Non-Word Dictation Test (Marinelli et al., in preparation) and the Lexical Spelling test (Nardacchione et al., submitted). Test procedures are described in Supplementary Appendix A.

#### 2.2.3 Assessment of numerical and computational skills

Numeracy and computation skills were examined with the tests of Number dictation, Reading numbers, Arithmetic facts, Mental calculation, Approximate Computation, Number Transcription of the LCS-SUA tests (Montesano et al., 2020) and with the tests of Written Calculations, Calculations in Mind, Transcription of Numbers, Find the Major (Letters), Find the Major (Digits), Tables and Simple Arithmetic Facts (Marinelli et al., in preparation). Test procedures are described in Supplementary Appendix A.

#### 2.2.4 Experimental test

**FIGURE 2:**
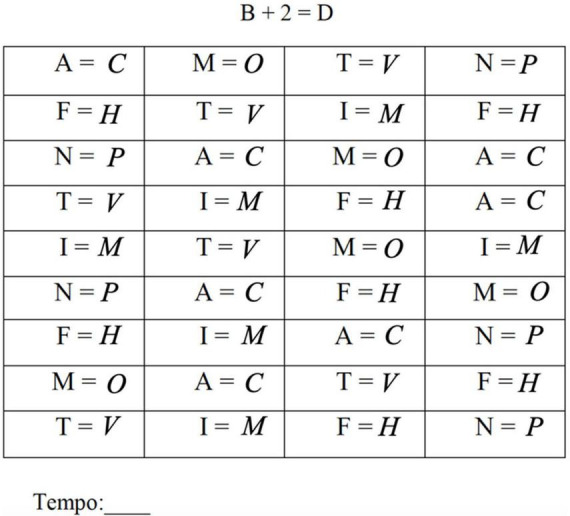
Example of a completed matrix of the experimental test.

The “Experimental Test” (Marinelli et al., 2021), a paper-and-pencil test of 22 matrices (9 x 4) of 36 exercises each, was used to test the automatization deficit. In each exercise, the participant sees a target letter and must produce the letter which is two letters ahead in the alphabet (e.g., A + 2 = C; D + 2 = ?). The target letters were always the same so that participants could switch from a slower performance due to the application of the counting algorithm (A + 2 = C) to a more automated one based on retrieving the solution (C) from memory from observation of the target letter (A). Figure 2 shows an example of a completed Experimental test matrix.

In the A1 to A20 matrices, the letters tended to repeat to allow for testing learning, while the matrices B1 and B2 included different target letters from those in the A matrices to allow for testing generalization. The test was preceded by a pre-test matrix of 8 items (2 ×4) based on different letters. The test was given on a single day but with a brief pause between the matrices A10 and A11. Every time the participant finished performing the exercises within a matrix, the execution time was recorded. The number of errors and the execution times were recorded for each matrix.

### 2.3 Data analysis

First, we compared adults with SLD and controls on the reading, spelling, and math tests by t tests for independent samples. Due to the presence of multiple comparisons (*N* = 21), the *p* < 0.05 value was adjusted to a α value based on the Bonferroni correction (yielding a reference value of *p* = 0.002).

The instance theory of automatization of Logan (1988, 1992) predicts that learning follows a power function and that the shape of the learning curve is determined by practice, and it is closely related to the shape of the response time distribution. The power function is:


T=a*x+bc

where T indicates time, a and b are scaling parameters (a is the asymptote reflecting an irreducible limit on performance, and b is the difference between initial and asymptotic performance), x is the amount of practice and c, the exponent, is the rate of learning (with higher values indicating steeper slopes). The shape of the curve varies as the exponent varies.

Initially, we employed Equation 1 to model individual data using a least-squares approach. This allowed us to assess whether performance improved with practice, following a power function consistent with the model. The asymptote, *a*, was constrained to be no lower than the minimum time each observer spent on the matrices, regardless of session number. Successively, we estimated the three main parameters of the individual “by using Matlab 2020a (the MathWorks Inc., Natick, MA, USA)”: a, b, and c. Moreover, for each fit, we considered the R^2^, a measure of the variance explained, with higher R^2^-values indicating better fits. While individual power fits were generally good, some children exhibited irregular learning curves, leading to low individual R^2^ values. We applied an R^2^ threshold of 0.30 (Marinelli et al., 2021). As reported in the participant section, 33 participants showed somewhat irregular learning curves and low individual R^2^ values or abnormal exponents and were eliminated from the sample. The final median R^2^-values were 0.74 (ranging from 0.30 to 0.97) for the group with SLD (*N* = 59) and 0.74 (ranging from 0.31 to 0.95) for the control sample (*N* = 143).

One-way ANOVAs were conducted with the group (SLD vs. controls) as the between-subjects factor and the following parameters of the power curves at the Experimental test as the dependent variable: scaling value a, exponential b, asymptote c, and R^2^. Significant interactions were explored with Bonferroni’s *post-hoc* test. These statistical analyses (and the following) were conducted employing the software Statistica 8.0.550 (StatSoft. Inc., Tulsa, United States).

Then, a series of repeated measures ANOVAs were separately carried out using matrix execution times (total number of sec to perform each matrix) and accuracy (number of errors per matrix) as dependent variables. To investigate the effect of learning across experimental trials, a repeated-measures ANOVA using learning trial (from A1 to A20 matrix) as a within-subjects factor and group (participants with SLD vs. controls) as a between-subjects factor. To investigate the generalization of learning, performance at the first matrix (A1), the last one (A20) and the first matrix with new stimuli (B1) were compared through an ANOVA with condition (A1, A20, B1) as within-subjects factor and group (adults with SLD vs. controls) as between-subjects factor. Note that power fits are expected for the time measures, while no explicit prediction of the curve of learning is made in the case of accuracy, which was analyzed only as a control measure (these analyses are presented in Supplementary Appendix B). Sixteen participants with subclinical learning disorders were excluded from these analyses.

The same analyses described above were replicated with the type of deficit (“Poor procedural”, “Poor instance”, and “Control” groups) as a between-subjects factor. On these groups, we run a set of analyses like those carried out on the overall sample of participants. First, we tested the power fits on the individual and group data; furthermore, one-way ANOVAs were conducted on the power curve parameters of the three groups. Secondly, we examined the learning trial effect (A1–A20) separately on response times and accuracy. Finally, the generalization effect (A1 vs. A20 vs. B1) was examined separately on response times and accuracy. The analyses for the accuracy measures are presented in Supplementary Appendix B.

## 3 Results

### 3.1 Characteristics of the sample

The control sample consisted of 143 young adults with typical development (M: *N* = 32 and *F*: *N* = 111, mean age: 20.79 years, SD: 3.11; schooling in mean years = 16.3; *SD* = 1.9; Raven SPM mean score = 45.48, *SD* = 9.08). The experimental sample consisted of 59 young adults with specific learning disorders (*M*: *N* = 12 and F: N = 47, mean age: 20.98 years, *SD*: 3.04; schooling in mean years = 16.8; *SD* = 1.3; Raven score mean = 43.21, *SD* = 5.55). Experimental and control groups did not differ in sex (X^2^ = 0.10), age (*t* = 0.32, *p* = 0.75), and Raven’s SPM score (*t* = 1.53, *p* = 0.13). Table 1 presents the socio-demographic variables of the SLD and control groups.

**TABLE 1:** Socio-demographic variables of the sample in study 1.

	Adults with SLD		Controls			
	Mean	SD	Mean	SD	*t*-test	*p*
Age (years)	20.98 (range: 19-25)	3.04	20.79 (range: 18-41)	3.11	*t* = 0.32	0.75
Raven SPMs	43.21	5.55	45.48	9.08	*t* = 1.53	0.135
Gender	47 F, 12 M		111 F, 32 M		x^2^ = 0.75	0.10

We compared adults with SLD and controls on the reading, spelling, and math tests by *t* tests for independent samples. The performance of the experimental and control group in standardized tests is presented in Table 2 in terms of z-score values.

**TABLE 2:** Means and SDs at the LCS-SUA battery of the adults with learning disorders and controls.

	Adults with SLD		Controls				
	Mean	SD	Mean	SD	*t*-test	*p*	d Cohen
Reading comprehension	−1.55	1.11	−0.93	1.09	2.95	0.004	0.56
Text reading (errors)	−4.02	3.09	−0.74	1.08	7.54	0.000*	1.73
Text reading (syll/sec)	−1.14	1.14	−0.21	0.86	4.83	0.000*	0.97
Word reading (errors)	−1.78	2.14	0.00	0.72	5.97	0.000*	1.37
Word reading (speed)	−1.10	1.01	−0.06	0.89	5.61	0.000*	1.11
Non-word reading (errors)	−1.80	1.90	−0.17	0.81	5.95	0.000*	1.32
Non-word reading (syll/sec)	−0.97	0.81	−0.11	0.97	4.90	0.000*	0.93
Lexical decision (ASC)	−1.47	1.89	−0.24	1.08	4.28	0.000*	0.90
Text dictation (errors)	−1.00	1.66	0.04	0.89	4.22	0.000*	0.89
Long HF word dictation	−0.97	3.22	0.18	0.71	2.76	0.006	0.63
Long LF word dictation	−1.41	2.17	−0.52	−0.52	2.90	0.004	0.63
Long HF word dictation (ASC)	−0.99	2.02	0.26	0.58	4.61	0.000*	1.05
Long LF word dictation (ASC)	−0.72	1.71	0.26	0.60	4.15	0.000*	0.93
Number dictation (tot. err.)	−0.18	1.06	0.48	0.54	4.24	0.000*	0.90
Reading numbers (errors)	−1.79	2.85	0.12	0.85	4.99	0.000*	0.82
Reading numbers (tot sec)	−1.73	2.31	−0.40	1.22	3.90	0.000*	1.13
Arithmetic facts	−2.13	1.37	−1.06	1.12	4.49	0.000*	0.90
Mental calculation (corr. resp.)	−1.34	0.88	−0.82	0.82	3.19	0.002°	0.34
Mental calculation (tot sec)	−0.53	1.42	−0.11	1.14	1.75	0.081	0.62
Approximate computation (corr. resp.)	−0.85	0.83	−0.11	1.38	3.28	0.001*	0.59
Number transcription (in numbers)	−2.09	1.48	−0.43	1.90	4.96	0.000*	0.93

The data are z-scores, with negative scores indicating pathological performance. Group comparisons were carried out by *t* test for independent samples with an α adjusted for multiple comparisons of 0.002 by Bonferroni correction. NC, normal condition; ASC, articulatory suppression condition; HF, high frequency, LF, low frequency. *p < 0.002; °*p* = 0.002.

As reported in Table 2, participants with SLD performed less well than typically developing adults in all tests, except in Mental calculation (speed). Also, group differences in Text comprehension, and Long HF word and Long LF word writing fell short of significance after correction for multiple comparisons. Z-scores highlighted most impaired performance in Text, Words and Pseudowords Reading, Lexical decisions, Arithmetic fact, Number transcription and Number reading tests.

### 3.2 Learning acquisition on the experimental test

#### 3.2.1 Power fits

**FIGURE 3:**
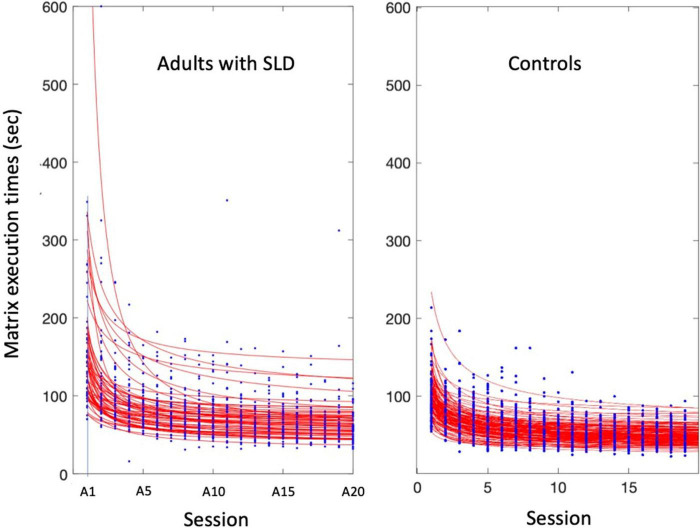
Power fits applied to the median performance of young adults with SLD and controls in study 1.

The individual fits of the data of participants with SLD and control participants are presented in Figure 3. An inspection of the figure indicates that the power law of learning is well represented by these data.

**FIGURE 4:**
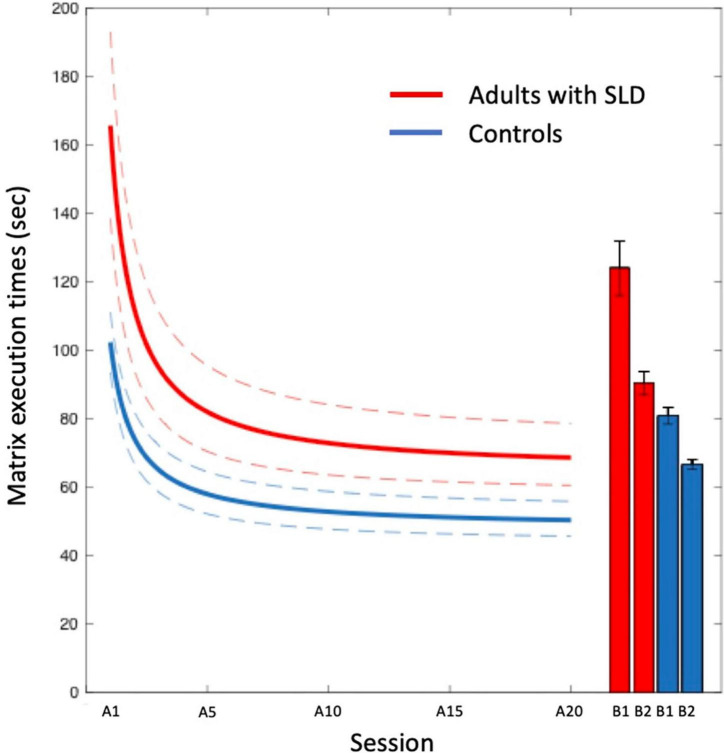
Power fits for the group of adults with SLD (solid red line) and controls (solid blue line) in the 20A sessions with 95% confidence intervals (dashed lines). Data are fit with a power function (see section 2). The parameter fits were *a* = 107, *b* = −85, and *c* = 57 for the group with SLD; *a* = 58, *b* = −78, and *c* = 42 for the control group. The histograms indicate the execution times for the novel (B) matrices (with standard errors).

Figure 4 report the power fits obtained by the two groups of participants (median of individual data of SLD and control participants) for response times across the 20A matrices, along with the 95% confidence intervals. Execution times reduced with practice according to the power law in both groups (*R*^2^ = 0.74) with the following mean global parameters: *a* = 106.70, *b* = −0.85, *c* = 56.55 for the group with SLD and *a* = 58.38, *b* = −0.78, *c* = 42.41 for the control group.

Figure 4 also shows the performance of the two groups of participants in the novel (B) matrices (and the standard errors). Note that performance on the B1 matrices is considerably worse than that on the last learning trial (A20), indicating little generalization of performance, as expected. Again, participants show an improvement already by a second presentation of these B matrices (B2). The comparison with the B matrices indicates little generalization of performance as expected based on instance learning.

The one-way ANOVAs between the two groups on the parameters of the power fits are presented in Table 3. The group effect for the scaling value “a” (initial performance) was significant [*F*_(1, 200)_ = 22.76, *MSE* = 97,519, *p* < 0.001, η*_*p*_*^2^= 0.10]: adults with SLD (106.70 ± 8.52 s) showed higher “a” values than controls (58.38 ± 5.47 s). The analysis on parameter “c” (asymptote) revealed that adults with SLD (56.55 ± 1.63 s) showed higher values for this parameter compared to the control group [42.41 ± 1.04 s; *F*_(1, 202)_ = 52.99, *MSE* = 8,351.1, *p* < 0.001, *η_*p*_*^2^ = 0.21]. In contrast, no differences emerged concerning the “b” exponent and R^2^, which were similar in the two groups.

**TABLE 3:** One-way ANOVAs on the power curve parameters of the experimental test in study 1.

	Adults with SLD		Controls					
	Mean	SD	Mean	SD	df	*F*	*p*	*η^2^*
a scaling value	106.70	8.53	58.38	5.47	1,200	22.76	<0.001	0.102
b exponential	−0.84	0.05	−0.77	0.03	1,200	1.42	0.23	0.007
c asymptote	56.54	1.63	42.40	1.04	1,200	52.99	<0.001	0.209
R^2^	0.68	0.02	0.71	0.01	1,200	1.34	0.25	0.006

#### 3.2.2 Effect of learning repetition (execution times)

The ANOVA on matrix execution times highlighted the significance of the main effect of group [*F*_(1, 200)_ = 70.03, *MSE* = 413,805, *p* < 0.001, *η_*p*_*^2^ = 0.26]: the control participants (58.11 ± 1.43 s) were faster to solve the task compared to the adults with SLD (80.37 ± 2.23 s). The main effect of the learning trial was significant [*F*_(19, 3800)_ = 143.15, *MSE* = 54,678, *p* < 0.001, η*_*p*_*^2^ = 0.42], indicating a learning effect across the 20 repetitions. With practice, matrix execution times decreased from A1 (126.50 ± 5.06 s) to A20 (53.87 ± 1.06 s.), i.e., by about 73 s (*p* < 0.043). The group by learning interaction trial was significant [*F*_19, 3800)_ = 13.72, *MSE* = 5,242, *p* < 0.001, η*_*p*_*^2^ = 0.06]. Results showed a larger decrease in matrix execution times with practice in adults with SLD (about 96 s from A1 to A20, with an average reduction per trial of 5.03) than in controls (about 50 s from A1 to A20, average reduction per trial = 2.61 s). Moreover, results showed a difference between groups: SLD participants were slower than control participants in all trials (at least *p* < 0.05) except for A13, A18 and A20, in which comparisons between groups did not reach statistical significance.

The ANOVAs on accuracy scores are reported in Supplementary Appendix B1.1.

#### 3.2.3 Testing generalization to new target stimuli (execution times)

**FIGURE 5:**
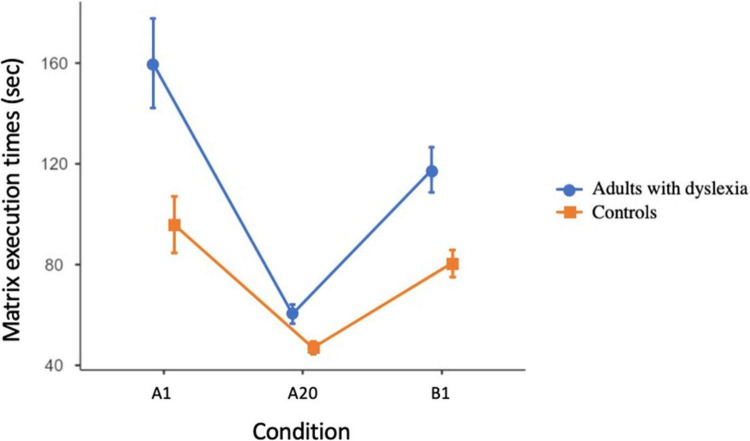
Generalization of learning in the Experimental Test in adults with SLD and controls in Study 1. Error bars indicate confidence intervals. A1: initial learning test; A20: last learning matrix; B1: first matrix with different target letters.

The “*instance theory of automatization*” by Logan (1988, 1992) predicts that there will be no (or little) generalization when new target stimuli different from the ones subjected to practice are used. Figure 5 shows the generalization to new stimuli in the two groups. The main effect of the group factor was significant [*F*_(1, 200)_ = 65.51, *MSE* = 191,295 *p* < 0.001, η*_*p*_*^2^ = 0.24]: adults with SLD (113.80 ± 4.06 sec) were slower than controls (74.72 ± 2.60 s). The effect of the condition factor was significant [*F*_(2, 40)_ = 147.76, *MSE* = 228,640, *p* < 0.001, η*_*p*_*^2^= 0.42]: performance in the B1 presentation (102.42 ± 3.04 s) was much slower than the performance at the A20 matrix (53.87 ± 1.06 s) but faster than the performance in the A1 (126.50 ± 5.05 s) presentation (about 24 s, *p* < 0.001). The group by condition interaction was significant [*F*_(2, 400)_ = 14.574, *MSE* = 22,552, *p* < 0.001, η*_*p*_*^2^ = 0.06]. The group difference was significant for A1 (*p* < 0.001) and B1 conditions (*p* < 0.001), with slower performance in adults with SLD compared to controls but not in A20, in which the two groups showed similar response times (*p* = 0.63).

The ANOVAs on accuracy scores are reported in Supplementary Appendix B1.2.

### 3.3 Learning “instances” according to the type of deficit

#### 3.3.1 Characteristics of the “Poor instance,” “Poor procedural,” and control groups

Overall, we obtained a “Poor instance” group of 24 participants, a “Poor Procedural” group of 22 participants, and a “Control” group of 132 participants (for characteristics of these groups see the Results section).

The “Poor instance” group consisted of 24 participants (*F* = 18, *M* = 6; mean age = 21.70 years, *SD* = 5.51; schooling in years: Mean = 17.0, *SD* = 1.4; Raven Score: Mean = 41.47, *SD* = 6.06); the “Poor Procedural” group consisted of 22 participants (*F* = 21, *M* = 1; mean age Mean = 21.33 years, SD = 5.43; schooling in years *M* = 16.9, *SD* = 1.8; Raven Score Mean = 44.78, *SD* = 3.74); the “Control” group of 132 participants (*F* = 100, *M* = 32; age *M* = 19.96, *SD* = 2.30; schooling in years M = 16.2, SD = 1.9; Raven Score Mean = 48.18, SD = 3.97). The groups did not differ in age [*F*_(2, 89)_ = 1.98, *p* = 0.11] or schooling [*F*_(2, 89)_ = 0.56, *p* = 0.63].

#### 3.3.2 Power fits

**FIGURE 6:**
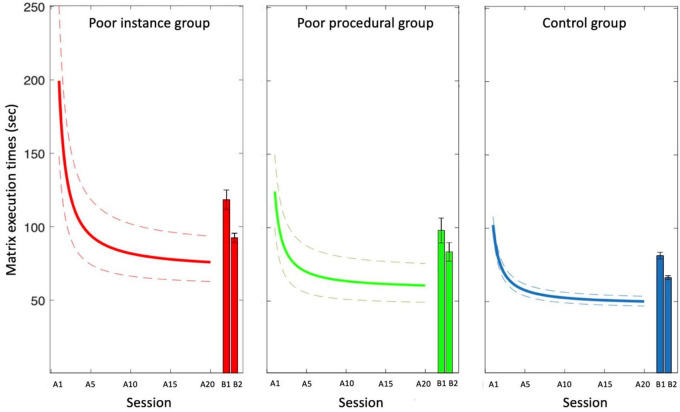
Learning effects in the Experimental Test in the “Poor instance,” “Poor procedural,” and “Control” groups of participants. Data are fit with a power function (see section 2) with the following parameters: *a* = 139, *b* = −77, and *c* = 56 for the “Poor instance” group; *a* = 71, *b* = −79, and *c* = 51 for the “Poor procedural” group; *a* = 59, *b* = −77, and *c* = 42 for the control group. The dashed lines represent the 95% confidence intervals. The histograms indicate the execution times for the novel (B) matrices.

The fits applied to the median of the data of each group are presented in Figure 6. Execution times reduced with practice according to the power law in all groups (parameters are reported in the legend of the figure).

The one-way ANOVAs on the power curve parameters are presented in Table 4. The ANOVA on scaling parameter “a” revealed a significant difference among groups [*F*_(2,175)_ = 14.45, MSE = 65817.1, *p* < 0.001, η*_*p*_*^2^ = 0.14]: the participants in the Poor instance group (139.09 ± 13.77 s) showed higher scores than the those in the Poor procedural (71.39 ± 14.38 s; *p* = 0.002) and Control (58.58 ± 5.87 s; *p* < 0.001) groups. Poor procedural and control group did not differ between them (*p* = 1.00). The ANOVA on scaling parameter “c” (asymptote) revealed a significant difference among groups [*F*_(2, 175)_ = 22.21, MSE = 2636.1, *p* < 0.001, η*_*p*_*^2^ = 0.20]: the participants in the Poor instance (56.48 ± 2.22 s) group showed higher values compared to those in the Control group (42.00 ± 0.94 s; *p* < 0.001); the participants in the Control group showed lower scores compared to those in the Poor procedural group (51.47 ± 2.32 s; *p* < 0.001). The Poor instance group showed similar values of asymptote compared to Poor procedural group (*p* = 0.326). The one-way on parameter R^2^ revealed a significant difference among groups [*F*_(2,175)_ = 3.493, MSE = 0.097, *p* = 0.032, η*_*p*_*^2^ = 0.03]: the Poor instance (0.63 ± 0.03) group showed lower determination coefficients compared to the Control (0.72 ± 0.01, *p* = 0.065) group but similar to the Poor procedural group (0.66 ± 0.03, *p* = 1.00); the Poor procedural group showed similar values than the Control group (*p* = 0.33). For the exponential “b,” the effect of the group was not significant.

**TABLE 4:** One-way ANOVA on the power curve parameters of the experimental test in study 2.

	Poor instance group		Poor procedural group		Control group					
	Mean	SD	Mean	SD	Mean	SD	df	*F*	*p*	η^2^
a Scaling value	139.09	13.77	71.39	14.38	58.58	5.87	2,175	14.45	<0.001	0.142
b Exponential	−0.76	0.07	−0.79	0.07	−0.77	0.03	2,175	0.94	0.95	0.000
c Asymptote	56.48	2.22	51.47	2.32	42.00	0.94	2,175	22.21	<0.001	0.202
R^2^	0.63	0.03	0.66	0.03	0.72	0.01	2,175	3.49	0.032	0.038

Comparison of “Poor procedural,” “Poor instance,” and “Control” groups.

#### 3.3.3 Effect of learning repetition (execution times)

The ANOVAs on matrix execution times highlighted the significance of the main effect of group [*F*_(2, 175)_ = 34.30, MSE = 178,582, *p* < 0.001, η*_*p*_*^2^ = 0.28]: the Control group participants (57.68 ± 1.40 s) were faster compared to both participants in the Poor procedural (70.26 ± 3.43; *p* = 0.002 s) and Poor instance (86.25 ± 3.29; *p* < 0.001 s) groups. Moreover, the Poor procedural group participants were faster than those in the Poor instance group (*p* = 0.002).

The main effect of the learning trial was significant [*F*_(19, 3325)_ = 93.67, *MSE* = 36,502, *p* < 0.001, η*_*p*_*^2^ = 0.34], indicating a significant learning effect across the 20 trials: execution times decreased with practice from A1 (133.50 ± 6.89 s) to A20 (54.94 ± 1.33 s) by about 79 s (*p* < 0.030).

The group by learning trial interaction was significant [*F*_(38, 3325)_ = 8.87, *MSE* = 3,458, *p* < 0.001, η*_*p*_*^2^ = 0.09]. In particular, results showed a significant decrease in response times with practice in all groups, but larger in the Poor Instance group (about 123 s from A1 to A20, average of reductions for each additional learning trial = 6.48), compared to the Poor procedural group (about 62 s from A1 to A20, average decrease for each additional learning trial = 3.29), and the Control (about 50 s from A1 to A20, average decrease for each additional learning trial = 2.63) group. Participants in the Control group reduced their times by 43 s from A1 (Mean 96.29 s) to A11 (53.06 s) and 6.75 s from A11 to A20 (46.30). The participants in the Poor instance group reduced their times by 98.87 s from A1 (Mean = 187.20 s) to A11 (88.33 s) and by 6.75 s from A11 to A20 (64.08 s). The participants in the Poor procedural group reduced their times by 52 s in solving the matrices from A1 (Mean 117 s) to A11 (64.04 s) and 9.59 s from A11 to A20 (54.45 s).

Moreover, results showed a difference among groups: the participants in the Poor instance group were slower compared to those in the Poor procedural group only in A1 and A2 presentation (*p* < 0.001 for both comparisons) and compared to the Control group in the first 10 trials (at least *p* < 0.008). The “Poor procedural” and “Control” groups did not differ between them.

The ANOVAs on accuracy scores are reported in Supplementary Appendix B2.1.

#### 3.3.4 Testing generalization to new target items (execution times)

The main effect of group was significant [*F*_(2, 175)_ = 27.66, *MSE* = 73,664, *p* < 0.001, η*_*p*_*^2^= 0.24]: the “Poor instance” group participants (123.22 ± 6.08 s) were slower to solve the task compared to both those in the “Poor procedural” (89.84 ± 6.35 s; *p* < 0.001) and “Control” (74.62 ± 2.59 s; *p* < 0.001) groups. The “Poor procedural” and “Control” groups did not differ between them. The condition effect was significant [*F*_(2, 350)_ = 97.96, *MSE* = 147,418, *p* < 0.001, η*_*p*_*^2^ = 0.35]. There was a significant decrease in execution times (about 78 s) between A1 and A20 (*p* < 0.001) and a significant increase in the condition with new stimuli (B1) compared to the A20 presentation (about 44 s., *p* < 0.001). The performance in the B1 presentation (99.25 ± 3.22 s) was much slower than the performance at the A20 matrix (54.94 ± 1.33 s) but faster than that in the A1 (133.50 ± 6.89 s) condition (about 34 s, *p* < 0.001).

**FIGURE 7:**
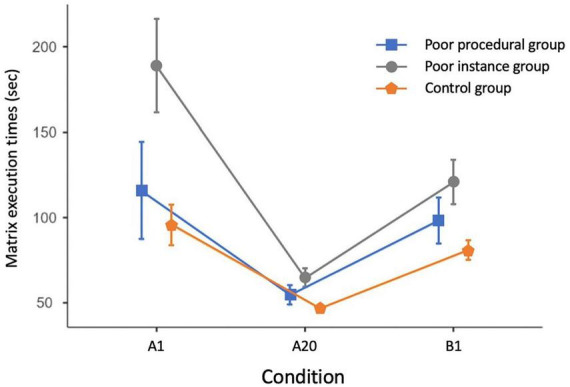
Generalization effect in the Logan Test in “procedural,” “instance,” and control participants in Study 2. Error bars indicate confidence intervals. A1: initial learning test; a20: last learning matrix; b1: first matrix with different letters.

The group by condition interaction was significant [*F*_(4, 350)_ = 9.75, *MSE* = 14,670, *p* < 0.001, η*_*p*_*^2^ = 0.10; see Figure 7]. Response times decreased with practice from A1 to A20 in all groups, but the difference was larger in the “Poor instance” group (*p* < 0.001; about 123 s) compared to the “Poor procedural” (*p* < 0.001; about 62 s) and “Control” (*p* < 0.001; about 50 s) groups. The three groups showed a significant and similar increase in times in the condition with new stimuli (B1) compared to the A20 (“Poor instance” group: about 54 s, *p* < 0.001; “Poor procedural” group: about 43 s, *p* = 0.008; and “Control” group: about 35 s, *p* < 0.001). Finally, performance in the B1 presentation was much faster than in the A1 only for the “Poor instance” group (about 68 s, *p* < 0.001).

The ANOVAs on accuracy scores are reported in Supplementary Appendix B2.2.

## 4 Discussion

The study examined the performance of college students in a novel task in which the participant must learn to apply a new rule to already acquired information (alphabet). With practice, participants became progressively faster and, as expected, the learning progress followed a power function curve. Execution times substantially slowed down when the ability to respond was tested by changing the target stimuli in matrix B1. This pattern indicates the substantial absence of generalization of learning consistent with the idea that this occurs through acquiring specific instances (Logan, 1988, 1992). This pattern of results is generally consistent with what Marinelli et al. (2021) observed with a developmental sample.

The results revealed substantial differences between participants with SLD and controls. SLD participants started their performance at a much lower level than the controls but made appreciable improvements with exercise even though their “endpoint” of training-related learning (i.e., asymptote) was significantly slower than controls. The control participants started with a more effective (i.e., faster) performance and increased their performance by reaching a significantly lower asymptote. It should be noted how the differences between the two groups are related to these two scaling values, while there were no differences in the learning trend; in other words, the trend of performance improvement in the two groups of participants had the same power curve coefficient. Consistent with the hypotheses, as a group, participants with SLD had a higher asymptote (i.e., slower times) at the end of learning, index of difficulty in efficiently forming single representations of the solution of various target stimuli (or instances). This finding is in keeping with the idea formulated based on the “*multilevel model of learning*” by Zoccolotti et al. (2020a,b) that difficulty or inefficiency in forming individual memories may contribute to overall performance in school learning tasks, such as reading, spelling, and computation.

However, as presented in the introduction, there are alternative views to explain the deficit of automatization. According to the “*Procedural deficit hypothesis*” (Ullman, 2004; Ullman et al., 2020), automatization is achieved through procedural (not declarative) processes. To test these alternative interpretations, participants were divided into two sub-groups, one failing in instance-based types of tests (“Poor instance” group) and one failing in procedural tests (“Poor procedural” group) across the three domains tested (reading, spelling, and mathematics). This allowed us to put this contrast to an empirical test.

Results indicated marked differences among the three groups of participants analyzed. The participants in the Poor instance group had significantly slower initial performance (as indicated by a significant difference in scaling value a) and a higher endpoint (i.e., slower times at the end of the training period) than the participants in the Control group but also of participants of the Poor procedural group. These results are consistent with the hypothesis that one of the relevant components in accounting for performance in complex behavioral tasks is the ability to acquire and consolidate single event memories (instances) and that this component is domain-independent (i.e., it influences reading, spelling, as well as math performance) (Zoccolotti et al., 2020b). Thus, according to this interpretation, the observed results indicate an association between the difficulty in constructing individual memories (instances) in a (relatively) novel task and performance in established behaviorally in different domains which rest upon the knowledge of item-based information, such as writing or judging (through reading) ambiguous words and recalling arithmetic facts and lexical representation for spelling or performing orthographic judgment tasks (i.e., reading) with irregular words.

Participants in the Poor procedural group were defined by difficulties with at least one procedure in one of the target behaviors (i.e., reading, writing, or computation). Although exhibiting this difficulty, these participants showed a performance in the learning task that was very similar (although not identical) to that of the participants in the “Control” group; further, they also showed similar performance in the expected failure to generalize to novel stimuli. These results also appear consistent with the predictions of the “*Multilevel model of learning”* (Zoccolotti et al., 2020a,2021b). Accordingly, a procedural-type deficit has a relevant specific effect but does not impair the ability to perform tasks requiring item-based information. Therefore, according to this interpretation, these participants can use information related to “instances” to partially compensate for their reading, spelling, or calculation difficulties.

The presence of different learning endpoints is consistent with the idea that participants with SLD (particularly those with poor instance performance on clinical tests) have difficulty efficiently forming single representations of the solution of various target stimuli (or instances). By contrast, it is more complex to understand why adults with SLD perform differently at the beginning of training. An interesting theoretical interpretation has been proposed by Speelman and Kirsner (2006). According to these authors, even “*hypothetically new*” tasks nevertheless also require recall of skills previously acquired by the participants, and the performance observed is related to a mix of these two components.^3^ Indeed, even the experimental task here requires prior knowledge (related to knowing the alphabet). Thus, the initial task slowness observed in adults with SLD as a whole and in the participants of the Poor instance group can be thought of as partially related to the inefficiency in recalling alphabet information to integrate it with the demands of the new task. If this interpretation were correct, the stronger initial slowness and the higher asymptote would be two sides of the same difficulty in stably acquiring information in memory. Of course, this interpretation requires further investigation and should be considered, at present, as a working hypothesis to be subject to further experimental investigation.

Ideally, to contrast the impact of procedural- and instance-based deficits, it would be optimal to subdivide each group according to the type of learning disorder. Accordingly, one could contrast groups of participants with procedural deficits in either reading, spelling, or mathematics. However, in the present study, there were not enough participants to make such subdivisions, and this remains an object for future research. However, the participants with poor procedural skills behaved very similarly to the control participants independently of the learning domain. Thus, it seems unlikely that a procedural deficit in a specific learning domain would show a deviant result. Similarly, the group with poor instance-based skills included participants with different patterns of impairments across domains, and the potential role of domain specificity may only be ascertained by further research. Another limitation is the presence of participants who experience a co-occurring procedural deficit among poor Instance groups. Further studies with larger samples might be helpful to examine the impact of this concomitant deficit. Another note of caution refers to the generalizability of results to populations speaking languages with more inconsistent orthographies. Italian orthography, the object of the present study, is very consistent, such that a lower reliance on lexical procedure has been compared to more inconsistent orthographies (Marinelli et al., 2015, 2016, 2020, 2023). This could make the instance acquisition deficit in SLD participants less evident compared to other languages.

Even with these limitations, the results of the present study provide insights into the likely source of comorbidities across different learning disorders. It appears that both rule-based and instance-based processing contribute to the performance in standardized tests of reading, spelling and math, which may lead to the diagnosis of dyslexia, dysgraphia or dyscalculia. These learning disorders are sometimes present in isolated form but are also frequently associated. Thus, we need to understand both the source of dissociations and associations across such learning disorders. The present results indicate that a low ability to acquire and consolidate instances may be at the base of the comorbidity of learning disorders; by contrast, there was little indication that procedural processing would contribute to such comorbidities.

## Data Availability

The raw data supporting the conclusions of this article will be made available by the authors, without undue reservation.

## Footnotes

1 It should be considered that some typically developing adults may be classified in the “Poor instance” and/or “Poor procedural” group since the use of the other unimpaired strategies would allow them to compensate sufficiently for difficulties that they would not lead to a clinical diagnosis. For example, in a very consistent orthography such as Italian the compensative used of the unimpaired sublexical procedure might allow to reach an adequate reading and spelling performance also in presence of a lexical deficit. Similarly, some cases with a subclinical deficit in clinical trials and, therefore, not examined in SLD vs. typically developing participants comparison, may have fallen into one of three groups.

2 For example, the text and word reading and spelling tasks and the lexical decision might be solved through reliance on either lexical (i.e., instance retrieval) or sublexical procedure (i.e., application of the sublexical mapping procedure) and for this reason were not considered. Similarly, mental and written calculation tasks were not examined, as these tasks might be solved using either counting or computational procedures, as well as through the retrieval of arithmetic facts.

3 In a careful examination of this issue, Speelman and Kirsner (2006) conclude that *“…*in most cases, learning a new task involves continued practice on old skills. These are skills that have been practiced in the context of some other task. Learning a new task will also involve the development of new skills. These are skills that are required to fill the gap between the repertoire of old skills possessed by the trainee and the skills necessary to perform the new task. These new skills will involve both task-related skills and skills for integrating the functioning of old and new task-related skills” (Speelman and Kirsner, 2006, p. 55).
