# Supplementary material for: Impaired instance acquisition as a cause of the comorbidity of learning disorders in young adults
Source: Front Behav Neurosci. 2025 Jun 10;19:1560362. doi: 10.3389/fnbeh.2025.1560362 (PMC12186060; doi:10.3389/fnbeh.2025.1560362)
Supplement: Supplementary file 1 [file Presentation_1.pdf]

APPENDIX A

Reading Assessment

Reading Comprehension (LCS-SUA Battery, Montesano et al., 2020)

The experimenter asks the participants to read a text silently and answer 14 multiple-choice questions with four alternatives. The questions include both inferential-general questions and specific questions. The number of correct responses is the measure of reading comprehension. The test has no time limit; the participant always has the text at disposal and is free to recheck it if need. If the participant has second thoughts about the answer given, he can modify it. One point is awarded for each correct response.

Text Reading (LCS-SUA Battery, Montesano et al., 2020)

The participant reads the text passage from the LCS-SUA Battery (Montesano et al., 2020) aloud, aiming for speed and accuracy. The performance is assessed by calculating an error score and a reading speed measure (in syllables per sec).

Word and Non-word Reading Test (LCS-SUA Battery, Montesano et al., 2020)

The participant must read four lists of words from the LCS-SUA Battery. These lists vary in word length and frequency (high-frequency short words, low-frequency short words, high-frequency long words, low-frequency long words). The participant must read as quickly and accurately as possible. In addition, the participant reads two lists of non-words (short-long). Accuracy and reading speed (in syllables per sec) are recorded.

***Word and Non-word Reading with time pressure (Marinelli et al., in preparation*).**

Word Reading Accuracy

The participant must read one hundred words as accurately as possible with a 45-second time limit. Words were not morphologically complex nouns with a low-medium frequency (mean frequency = 24.8, SD = 16.5) and were, on average, eight letters long (SD = 1.1). The number of accurate responses in the allotted time is counted.

Non-word Reading Accuracy

The participant must read one hundred trisyllabic non-words as accurately as possible with a 45-second time limit. The non-words are meaningless to allow assessing sublexical reading. Non-words were

derived by words, changing 2-3 letters and were similar for length (mean = 8.0, SD = 0.8, $F < 1$). The number of accurate responses in the allotted time is counted.

Lexical Decision in Articulatory Suppression Condition (LCS-SUA Battery, Montesano et al., 2020)

The participant must cross out only words in a list of 30 high-frequency words and 30 pseudowords within a 1-minute time limit. The task is performed in articulatory suppression condition while the participant continuously repeats the syllable "*la*".

Same/different Judgement (Marinelli et al., in preparation)

The Same/different Judgement test (Marinelli et al., in preparation) consists of a total of 30 pairs formed by three-syllabic non-words some spelt the same and some differently for the substitution of one letter (for a stimuli description, see Zoccolotti et al., 2020a,b). The particularity of this test is that, in addition to not always being the same, the non-word pairs are written in different characters to ensure that the participant used sublexical coding and not just visual comparison. Participants must place a checkmark only next to the pairs with identical non-words as accurately as possible within a 45-second time limit. The number of accurate responses in the given time is counted.

Orthographic Judgment (Nardacchione et al., in press)

The participant must judge the correctness of the words by ticking the YES or NO options next to each word. Stimuli are taken by the Orthographic Judgment Test (Marinelli et al., 2017). The test consists of 6 cards, comprising 36 words each, totaling 216 stimuli. Among them, some have errors while others do not. Each card corresponds to an experimental condition: high-frequency regular words, low-frequency regular words, high-frequency irregular words with atypical transcription, low-frequency irregular words with atypical transcription, high-frequency irregular words with typical transcription, low-frequency irregular words with typical transcription. Errors in regular stimuli are not phonologically plausible and then detectable with both lexical and sublexical procedures. Fake versions of irregular words are phonologically plausible errors that can be identified only by lexical reading and the use of orthographic representations. Participants had a maximum time of 30 seconds for each card and had to respond quickly and accurately. The number of accurate responses in the given time is counted.

Spelling Assessment

Text Dictation Tests *(LCS-SUA Battery, Montesano et al., 2020)*

The examiner dictates a passage aloud by pausing where the scans are indicated, modulating the dictation rhythm according to the student's writing speed. The examiner does not give explanations, either before or during dictation, on words or phrases that may be difficult to understand. For understanding, the student will have to refer only to the context. Maximum two breaks are allowed. The examiner may repeat the term just dictated only in special cases, no more than two times. One point is awarded for each incorrectly written word. If a word is wrong in several parts, we consider only one error. The sum of all errors is the total score of the test. Punctuation errors and omissions are not considered in the total error score.

Word Dictation Test in Normal and Articulatory Suppression Conditions (LCS-SUA; Montesano et al., 2020)

The experimenter dictates eight lists of words. Two lists consist of high-frequency long words and two of low-frequency long words. These lists are presented in two conditions: normal and articulatory suppression. In the standard condition, the experimenter dictates the lists with a steady but flexible rhythm. In the articulatory suppression condition, the participant must repeat aloud and continuously the syllable "la" while writing the words dictated by the experimenter.

Word and Non-word Dictation (Marinelli et al., in preparation)

The test consists of spelling over dictation 60 low-frequency (mean = 5.15, standard deviation = 5.02) words and 20 pseudowords in articulatory suppression condition (i.e., while participants repeat aloud and continuously the syllable "la"). There are 20 regular words, 20 irregular typical words and 20 irregular atypical words. The four experimental conditions are matched for length (mean letters = 9.51, SD = 2), frequency (only for words), and ortho-syllabic difficulties (presence of doubles or clusters of consonants). Note that irregular words require lexical reading, while pseudowords sublexical processing. Regular words may be spelt with reliance to eigther procedure. The participant must listen to the dictated word and transcribe it on a blank sheet of paper in an articulatory suppression condition, i.e., write while repeating aloud and continuously the syllable. The number of correctly spelt words and pseudowords is scored.

Lexical Spelling (Nardacchione et al., submitted)

The same stimuli used for the Orthographic Judgement task are used in a dictation task, namely Lexical Spelling (Nardacchione et al., submitted). The number of correctly spelt words for each condition is scored.

Numerical and Computational Assessment

Number Dictation (LCS-SUA, Montesano et al., 2020)

The Number Dictation test (LCS-SUA, Montesano et al., 2020) consists of two lists of 10 numbers of varying complexity. The test assesses number syntactic and lexical knowledge as correct spelling involves the lexical elements (units, teens, tens, multipliers) and the syntactic rules (additive and multiplicative components). The participant must write down under dictation, in Arabic code, numbers read by the experimenter, cautioning that great care is required, especially in cases of fractions or powers. The number can be repeated only once, at the participant's request. About scoring, one point is awarded for each error, separately for each list. It is evaluated whether or not each dictated number is spelt correctly (so only one error is counted even if a number was wrong in several aspects). The total score is the sum of wrong responses made by the participant in both lists.

Reading Numbers (LCS-SUA, Montesano et al., 2020)

In the Reading Number test (LCS-SUA, Montesano et al., 2020), the participant must read aloud a list of 20 numbers presented in Arabic code of varying length and instructional complexity, allowing the assessment of number syntactic and lexical knowledge. Different classes of number vocabulary (units, teens, tens, multiplications) are included. Concerning scoring, for the accuracy parameter, 1 point is awarded for each number omitted or incorrectly read. Self-corrections are not considered errors.

Arithmetic Facts (LCS-SUA, Montesano et al., 2020)

The Arithmetic Facts test (LCS-SUA, Montesano et al., 2020) consists of 30 arithmetic facts assessing the level of automatization, i.e., the ability to quickly retrieve a result from memory without using computational procedures. The participant must solve simple operations within 3 seconds based on previous knowledge without calculating them. After 3 seconds, the experimenter explains that time has elapsed and moves on to the next item. A response after the 3-second time limit is considered invalid even if correct. For scoring, 1 point is assigned for each valid response (i.e., correct and within the time limit), and 0 for a response incorrect and/or given beyond the time limit.

Mental Calculation (LCS-SUA, Montesano et al., 2020)

The Mental Calculation test (LCS-SUA, Montesano et al., 2020) assesses the strategic aspects of oral calculus. The participant must perform ten operations as quickly and accurately as possible. The time allotted for each item is 30 seconds. For each operation, the time is measured from when the examiner finishes saying aloud the item. The examiner must report the answer given and the time taken for each task. If, after 30 seconds, the participant has not provided an answer for an operation, the examiner moves on to the next operation by marking "30" in the "Time" column and the item as incorrect. The accuracy score is the sum of the correct responses given to individual items within the 30-second limit; the time score is the total time, i.e., the sum of the response times to individual items (in seconds).

Approximate Computation (LCS-SUA, Montesano et al., 2020)

The participant is presented with a series of operations that are difficult to solve and is asked not to carry out the operation but to choose from three alternatives the one corresponding to the correct result. The participant has only one minute to take the test; so, it would be ineffective to perform the operations, while it will be appropriate to reason by order of magnitude. One point is awarded for each correct answer.

Number Transcription (LCS-SUA, Montesano et al., 2020)

In the Number transcription test (LCS-SUA, Montesano et al., 2020), ten numbers are presented as words, and the participant must transcribe the ten numbers into numerals. The test examines the presence of syntactic and lexical errors. The test has no time limit. For scoring, 1 point is assigned to each correct response and 0 to each incorrect or omitted response.

Written calculation (Marinelli et al., in preparation)

Each card in this test consists of six operations per category: six additions, six subtractions, six multiplications, and six divisions. The participant must perform the operations as accurately as possible using a procedural routine in a maximum time of 60 seconds each. The total number of accurate responses in the allotted time is counted.

Calculations in Mind (Marinelli et al., in preparation)

This test consists of 50 multiplications to be solved in mind as accurately as possible in a maximum time of 60 seconds. The multiplications are composed in this way: there is a single digit on one side and two

on the other, e.g., 5x32 or 9x27, to have the participant use computational procedures and not retrieve arithmetic facts from memory. The number of accurate responses in the allotted time is counted.

Transcription of Numbers (Marinelli et al., in preparation)

The test consists of 20 numbers written in words, for example, "seven thousand two hundred and three." The participant must read and transcribe the words into numbers (*i.e.*, 7203) within 60 seconds. The number of accurate responses in the given time is counted.

Find the Major (Letters) (Marinelli et al., in preparation)

The test consists of 20 pairs of numbers written as words, placed next to each other (e.g., A: "twenty-two thousand six hundred and fifty"; B: "thirty-seven thousand six hundred and twenty"). The participant must cross out the letter representing the higher number of the pair within 60 seconds. The number of accurate responses in the given time is counted.

Find the Major (Digits) (Marinelli et al., in preparation)

The test consists of 84 pairs of numbers written in digits, placed next to each other, and arranged in 3 different columns (e.g., A: 10,006.07; B: 10,006.009). The participant must mark the number representing the higher number of the pair within 60 seconds. The number of accurate responses in the given time is counted.

Tables (Marinelli et al., in preparation)

The test consists of 50 stimuli. The participant must perform the products of multiplication (arithmetical facts) as accurately as possible within a 60-second time limit. The number of accurate responses in the allotted time is counted.

Simple Arithmetic Facts (Marinelli et al., in preparation)

The operations in this test involve simple additions and subtractions to let participants retrieve arithmetic facts from memory, not by computational procedures. The test consists of 50 simple operations, which the participant must perform within a 60-second time limit. The number of accurate responses in the allotted time is counted.

APPENDIX B

B1. Analyses comparing adults with SLD and controls

B1.1 Effect of learning repetition (accuracy)

The ANOVA on errors highlighted the significance of the learning effect across the 20 trials ($F_{(19, 3762)} = 7.70$, $MSE = 13.32$, $p < 0.001$, $\eta p^2 = 0.04$): on the first trial (A1), errors were significantly higher compared to all successive trials ($p < 0.039$ for all comparisons), except for A2, A3 and A4 (at least $p = 0.06$). The number of errors in the A2 trial was higher compared to all successive trials (at least $p < 0.01$), except for A3 ($p = 1.000$). The number of errors in the A3 was higher compared to all successive (at least $p < 0.01$), except for the A4, A5, A6, A7, A10, and A11 (at least $p = 0.09$). The main effect of the group was significant ($F_{(1, 198)} = 22.43$, $MSE = 9589.41$, $p < 0.001$, $\eta_p^2 = 0.10$): adults with SLD (4.32 ± 0.60) made more errors than the controls (0.92 ± 0.39). The interaction between the group and learning trial was not significant ($F_{(19, 3762)} = 0.816$, $MSE = 1.43$, $p = 0.67$, $\eta p^2 = 0.004$).

B1.2 Testing generalization to new target stimuli(accuracy)

The main effect of the group was significant ($F_{(1, 198)} = 25.26$, $MSE = 1482.369$, $p < 0.001$, $\eta_p^2 = 0.11$): adults with SLD (4.59 ± 0.57) made more errors than the control participants (1.15 ± 0.37). The main effect of the condition factor was significant ($F_{(2, 396)} = 3.53$, $MSE = 23.444$, $p = 0.030$, $\eta p^2 = 0.01$): in A1, errors (3.22 ± 0.37) were significantly higher ($p = 0.010$) compared to the A20 (2.48 ± 0.36). Performance in the B1 matrix (2.91± 0.39) was intermediate and did not differ from either the A1 matrix ($p = 0.32$) or A20 ($p = 0.44$) matrix. The group by condition interaction was not significant ($F_{(2, 2, 396)} = 0.383$, $MSE = 2.366544$, $p = 0.681$, $\eta p^2 = 0.001$).

B2. Analyses comparing the Poor instance, Poor procedural and Control groups

B2.1 Effect of learning repetition (accuracy)

Results of accuracy showed a significant effect of learning across the 20 presentations ($F_{(19, 3287)} = 8.14$, $MSE = 11.66$, $p < 0.001$, $\eta p^2 = 0.04$). Post-hoc comparisons showed that in the A1 (3.82 ± 0.46), errors were significantly higher compared to all successive trials (from A4 3.34 ± 5.05 to A20 2.79 ± 0.44; $p < 0.029$ for all comparisons), except for A2 and A3 ($p = 1.000$). The number of errors in A2 was higher compared to all successive (126.50 ± 5.05; $p < 0.008$) except for A4 ($p = 1.000$). The number of errors in the A3 (126.50 ± 5.05) was higher compared to matrices after A7 (at least $p < 0.020$), except for A11 (the first one after the break).

The main effect of the group was significant ($F_{(2, 173)} = 14.34$, $MSE = 5374.10$, $p < 0.001$, $\eta_p^2 = 0.14$): the “Poor instance” group (6.10 ± 0.90) made more errors than the “Poor procedural” (1.86 ± 0.92; $p = 0.003$) and “Control” (0.86 ± 0.37; $p < 0.001$) groups. The comparison between the “Control” and “Poor procedural” groups was not significant ($p = 0.95$).

The group by learning trial interaction was significant ($F_{(38, 3287)} = 2.34$, $MSE = 3.35$, $p < 0.001$, $\eta p^2 = 0.02$). Instances participants make more errors compared to the control group in all trials (at least $p < 0.009$) and only in A18 compared to the Procedural Group ($p = 0.049$). Moreover, errors decreased in the earlier (first and second) presentation trial only for the "Poor procedural” and “Control” groups ($p < 0.034$). By contrast, for the “Poor instance” group, the errors were not reduced across trials. The participants in the control group reduced their errors of 0.55 in solving passing from A1 (Mean 1.40) to A11 (0.83) and of 0.106 passing from A11 to A20 (0.72). The instance group reduced their errors of 0.43 from A1 (Mean 6.52) to A11 (6.08), with no further reductions appreciable. The participants in the procedural group reduced errors of 1.54 from A1 (Mean 3.54) to A11 (2.00) and 0.63 from A11 to A20 (1.36).

B2.2 Testing generalization to new target items (accuracy)

The main effect of the group was significant ($F_{(2, 173)} = 19.17$, MSE = 1017.114, $p < 0.001$, $\eta_p^2 = 0.18$): the "Poor instance” group (6.92 ± 0.87) made more errors than the “Poor procedural” (2.36 ± 0.89; $p = 0.001$) and “Control” groups (1.04 ± 0.36; $p < 0.001$). The comparison between the “Control” and “Poor procedural” groups did not reach statistical significance (p = 0.54).

The condition effect was significant ($F_{(2, 346)} = 5.27$, MSE = 29.903, $p = 0.005$, $\eta_p^2 = 0.29$): Results indicated a significant decrease in errors with practice comparing A1 (3.82 ± 0.46) to A20 (2.79 ± 0.44, $p = 0.004$), while comparisons between B1 (3.71 ± 0.52) and A1 ($p = 0.880$) as well as B1 and A20 (p = 0.097) did not reach statistical significance.

The interaction between group and condition was significant ($F_{(4, 346)} = 2.697$, $MSE = 15.301$, $p = 0.030$, $\eta p^2 = 0.01$): the participants in the Poor instance group made more errors compared to those in the Poor procedural group in A20 and B1 presentation ($p < 0.015$ for both comparisons) and compared to the participants in the Control group in A1, A20 and B1 ($p < 0.008$ for all comparisons). The Poor procedural and Control groups did not differ in any conditions (p = 0.100).
